# A Study of Early-Stage Corrosion Behavior of AZ91 Alloy and MAO-Coated Alloy in 3.5% NaCl Solutions

**DOI:** 10.3390/ma15227909

**Published:** 2022-11-09

**Authors:** Yuxiang Liu, Xiaoting Liu

**Affiliations:** National Key Laboratory for Remanufacturing of China, Beijing 100072, China

**Keywords:** AZ91 alloy, MAO treatment, through pores, early-stage corrosion behavior, cathodic control

## Abstract

The early-stage (1 h) corrosion behavior of AZ91 alloy before and after microarc oxidation treatment in a 3.5% NaCl solution was revealed using open circuit potential, potentiodynamic polarization, electrochemical impedance spectroscopy, and the observations of corroded surfaces at different immersion time (5, 25 and 45 min). The coating offers excellent corrosion resistance for the substrate for 1 h immersion corrosion by serving as a physical barrier. For the alloy, pitting corrosion initiates easily, but propagates difficultly, due to the formation of oxides/hydroxides in the pits. By comparison, the localized corrosion of the coated alloy proceeds continuously via the through pores, and the newly formed corrosion products inside the pores are easily damaged. Based on the electrochemical results, the alloy exhibits quasi-uniform corrosion, and the coated alloy reveals localized corrosion, both of which are under cathodic control.

## 1. Introduction

Since the equilibrium potential of oxidation reaction of magnesium (Mg) metal is far below that of reduction reaction of H_2_O or H^+^, electrochemical redox reactions proceed spontaneously on Mg electrodes in aqueous solutions. The thermodynamic instability of Mg alloys and the impassivity of corrosion products in a relatively wide pH range are two main aspects that limit Mg industrial applications. Thus, it is essential to reduce the corrosion rate of Mg alloys before using them in aerospace, automotive, electronic products, and implants [1,2,3]. Alloying is a principal approach to improve the corrosion resistance of Mg by adding elements (always Al, Zn, Mn, RE, et al. [4,5]) and delicately adjusting their contents simultaneously. However, alloying with noble elements might enhance the micro galvanic corrosion of magnesium since the galvanic potential of Mg is also very negative (−1.6 V vs. SCE in seawater [6]). An alternative and practical approach to depressing the corrosion rate of Mg alloys is a coating that can immune corrosion by providing a protective barrier [7,8,9]. More importantly, various coatings can be fabricated on Mg alloys by tailoring coating parameters for different applications.

Among coating technologies capable of protecting metals, the micro-arc oxidation (MAO) technique with outstanding adhesion strength to Mg substrate has attracted increasing attention [10,11,12]. It is an environmentally friendly approach and is superior to traditional electroplating, which uses complicated plating potions [7]. Moreover, it is more flexible in treating irregular-shaped parts with respect to thermal coating and laser cladding [4]. Previous studies [13,14,15,16] reported that the MAO coating would delay the rate of corrosion attack during the initial period of corrosion, (several hours to tens of hours) but would be damaged by aggressive ions at the vulnerable sites (pores and cracks), especially for long term immersion tests or salt spray tests. The damage time usually depends on the properties of the coating (compositions and structure, et al.) and the testing environment (electrolyte and temperature, et al.). However, the overall compactness, in terms of porosity and other defect levels of MAO coatings, is the essential reason for the localized corrosion of the coated alloy [11,15] because the pores and cracks are corrosion-active sites where the coating ruptures easily. In addition, the corrosion products produced by the acute electrochemical redox reactions of the underlying Mg substrate cause stress inside the coating, leading to more defects and more severe damage. Moreover, the surface of MAO coating is also very rough, leading to a hydrophilic nature which is also responsible for the coating failure [17,18].

Although it is a consensus that the corrosion damage of MAO-coated Mg alloy is time dependent and the long-term corrosion process is well established, the early-stage corrosion characteristics, in terms of corrosion proceeding and corrosion type, are rarely explored. In fact, the early-stage corrosion morphology and electrochemical information can reveal how corrosion initiates and propagates before a coating’s breakdown while the corrosion is mature and monotonous in long-term experiments [19,20,21,22,23]. Therefore, an investigation (corrosion morphology and electrochemistry) on the early-stage corrosion process of Mg alloy and the MAO-coated alloy is essential to fully understand their corrosion behavior. In this study, we first prepared the phosphate ceramic MAO coating on AZ91 Mg alloy. Then, we examined the microstructure and corrosion morphology of the alloy and the coated alloy before and after they were immersed in the 3.5% NaCl solution for 1 h. Meantime, we compared the time-dependent electrochemical corrosion characterizations in 1 h. Finally, we revealed the early-stage corrosion mechanisms.

## 2. Materials and Methods

### 2.1. Samples

A commercial AZ91 Mg alloy sheet, 5 mm in thickness, was used in this study. The chemical compositions of the alloy are given in Table 1. Rectangular specimens (10 mm × 10 mm × 5 mm) were wire-electrode cut from the sheet. Then, all six faces of specimens were ground through successive grades of SiC papers up to 2000 grit, followed by ultrasonic degrease in acetone, washing in deionized water, and drying in cold air. The MAO coatings were prepared using a homemade pulsed power supply with a square waveform. The maximum output of the power supply is 1000 V/5 A. An aqueous electrolyte was prepared by dissolving reagent-grade sodium phosphate (10.5 g/L) and potassium hydroxide (1 g/L) in deionized water. The pH of the electrolyte was 12.76. The electrolyte temperature was kept at 15–20 °C using a water-cooling facility during the MAO treatment. A stainless steel (type 304) barrel of dimension ϕ 20 mm × 25 mm was used as the electrolyte container and cathodic electrode at the same time. The ground alloy samples were anodes and immersed in the electrolyte during MAO treatment. In this work, a constant voltage of 500 V with a frequency of 400 Hz and a duty cycle of 30% was applied to the samples. The total treatment lasted 15 min. After treatment, all specimens were placed in hot water (80–100 °C) for 10 min to wash the residual electrolyte inside the coatings. Finally, the MAO-coated samples were dried using cold air before microstructural characterization and electrochemical measurements.

### 2.2. Experimental Techniques

Morphology and microstructure characterization of AZ91 Mg alloy before and after MAO treatment was carried out by scanning electron microscopy (SEM, Quanta 200, FEG, FEI, Hillsboro, OR, USA), equipped with energy dispersive X-ray (EDS) analysis facilities. Before SEM observation, the MAO-coated samples were cut into two pieces by a wire-electrode cutting machine. Then, the cross section of one piece and the surface of alloy samples were ground to 2000 SiC grade, followed by finishing with 0.25 μm diamond paste. To mitigate the charging on the ceramic coating during SEM observation, the MAO-coated samples were sprayed and deposited by a 10 nm gold film. The EDS compositions excluded the Au element and then were reported.

The immersion tests were conducted in a 3.5% NaCl solution for different times (15, 25, and 45 min). Before immersion, all specimens were coated with lacquer (Stopper 45 MacDermid, Denver, CO, USA), leaving working areas of 1 cm^2^. After corrosion, the specimens were washed in deionized water without removing any corrosion products. The corroded specimens were sprayed with gold in a vacuum and then observed using SEM. The concentration of Mg^2+^ in the solution after immersion for the different times was measured by inductively coupled plasma optical emission spectroscopy (ICP-OES, Thermo Scientific™ iCAP™ 7400, Waltham, MA, USA). The pH values of the solutions before and after corrosion were measured by a PHSJ-3F Rex laboratory pH meter (INESA Scientific Instrument Co., Ltd., Shanghai, China).

Electrochemistry measurements were carried out using a Zahner-Ennium electrochemical workstation (Zahner, Gundelsdorf, Germany) in the 3.5% NaCl solution at room temperature. A three-electrode cell configuration, consisting of a working electrode (a sample), a Pt counter electrode, and an SCE reference electrode was used. The samples were coated with lacquer, and the exposed surface area of the working electrodes was 1 cm^2^. The open-circuit potential (OCP) of the alloy and the coating were recorded for 1 h to evaluate the equilibrium of the system. The potentiodynamic polarization experiment was conducted after OCP recording. The scans were performed in opposite directions starting from OCP at a rate of 1 mV/s in a range from −1 to 1 V vs. OCP. For both the alloy and the coated samples, at least three potentiodynamic polarization curves were obtained. Electrochemical impedance spectroscopy (EIS) was performed at a frequency range of 10^5^ Hz to 10^−2^ Hz, with a signal amplitude of 10 mV. Fitting curves were performed with the Zview software. For the experimental data statistics, three duplicates were conducted for the alloys before and after MAO treatment.

## 3. Results

### 3.1. Microstructure

**Figure 1:**
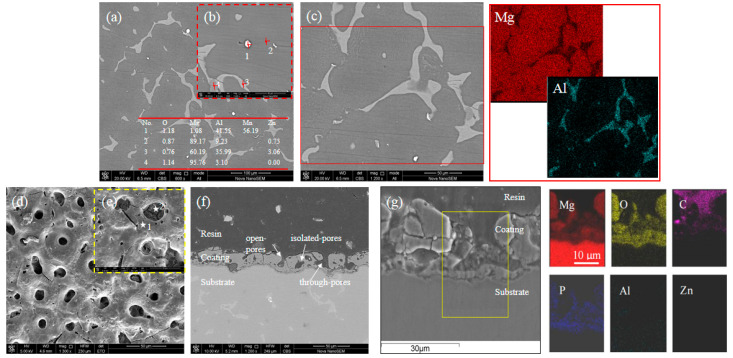
SEM images showing the microstructure of the AZ91 alloy before and after MAO treatment. (**a**) SEM image and (**b**,**c**) magnified SEM images for the as-received alloy; (**d**,**e**) Coating surface image; (**f**,**g**) Interface images for the coated alloy. The EDS mapping images correspond to the SEM images of (**c**,**g**).

Figure 1 shows the SEM images and EDS elemental distribution of the AZ91 alloy before and after MAO treatment. Figure 1a,b show that the as-received alloy is mainly composed of the α-Mg phase and the discontinuous β phase. In addition, the Al-Mn phase (41.55 wt.% Al, and 56.19 wt.% Mn at point 1 in Figure 1b) exists, and it appears white in the SEM images. The EDS point spectra confirm the β phase comprising 60.19 Mg, 35.99 Al, 3.06 Zn, and 0.76 O in wt.% (point 3). From the EDS mapping results in Figure 1c, Zn is absent because of its low amount, and the appearance of O indicates the oxidation of the alloy in the air. Oxidation also occurs on β phases and the amount of O of α phase and β phase approximates about 1 wt.%. Figure 1d,e show that the coating surface is characterized by pores with an average size of 10 μm and microcracks that always originate from the edge of pores and go through the adjacent pores. The average chemical compositions of the smooth surface (point 1 in Figure 1e) are 48.20 O, 16.41 P, 31.45 Mg, and 3.94 Al, indicating that Mg oxides/hydroxides and phosphates are the main constituents. The microstructure inside the pores is unlike the surface, which can be attributed to the formation of more Mg oxides (56.90 O, 1.22 P, 35.84 Mg, and 6.03 Al, see point 2 in Figure 1e), when intensive discharges took place in the pores [24,25]. Figure 1f,g show the cross-section images of the coated alloy, exhibiting a good coating/substrate adhesion. The overall thickness of the coating is 25–30 μm. Three types of pores can be observed: isolated, open, and through pores [10]. Figure 1g shows the morphology of a through pore that has been reported to be the vulnerable site where localized corrosion can be easily triggered. EDS elemental mapping images show that O and P are the main elements that constitute the oxides/hydroxides and phosphates.

**Figure 2:**
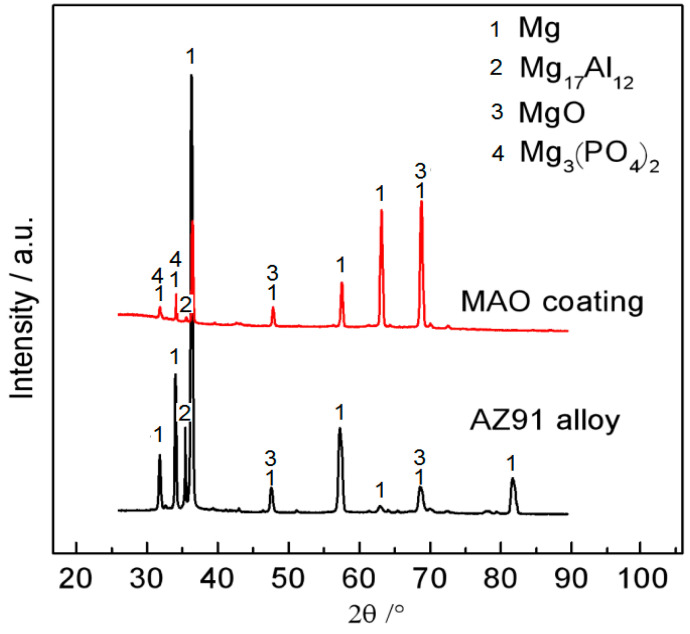
XRD patterns of the alloy and the coating.

Figure 2 shows the XRD spectra of the AZ91 alloy and the MAO-coated alloy. The two main phases can be identified for the as-received alloy: Mg and Mg_17_Al_12_. The oxide (MgO) phase is detected, but the Al-Mn phase cannot be detected, possibly because the latter one has a very small fraction and volume. By comparison, the Mg_17_Al_12_ phase is concealed by the coating materials that are composed of MgO and Mg_3_(PO_4_)_2_. The XRD results are consistent with the EDS results in Figure 1.

### 3.2. Electrochemical Measurements

**Figure 3:**
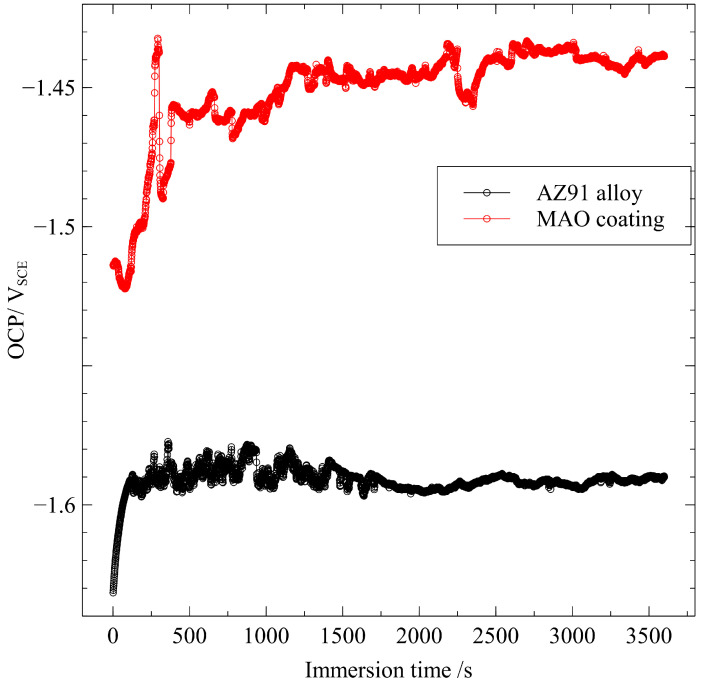
Time-dependent corrosion potential of the AZ91 alloy before and after MAO treatment for 1 h immersion in a 3.5% NaCl solution.

The open-circuit potentials (OCP), as a function of immersion time in Figure 3, show a similar evolution tendency for AZ91 alloy and MAO-coated alloy. The potential increases rapidly for 150 s and then fluctuates intensively from 150 to 1800 s. Finally, it reaches a relatively stable value after 1800 s. After 1 h immersion, the final potential for the MAO-coating alloy reaches a nobler value (−1.44 V_SCE_) with respect to the AZ91 alloy (−1.59 V_SCE_). Notably, the coated alloy always exhibits nobler OCP values than the alloy in the 3.5% NaCl solution. For the MAO-coated alloy, the coating can offer good protection for the underlying alloy before the coating’s breakdown. In such a case, the electrochemical reactions on the alloy are stifled. However, once the coating ruptures, the galvanic corrosion between the coating and the alloy is triggered and the underlying alloy serves as the anode due to its lower potential, aggravating the metal dissolution at the moment.

**Figure 4:**
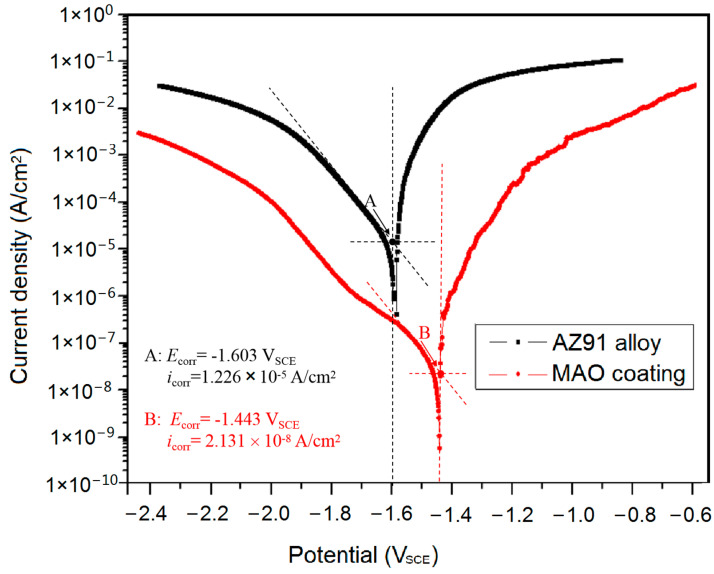
Polarization curves of AZ91 alloy, before and after MAO treatment after 1 h immersion in a 3.5% NaCl solution.

Figure 4 shows potentiodynamic polarization (PDP) curves for AZ91 alloy before and after MAO treatment after 1 h immersion in the 3.5% NaCl solution. Generally, corrosion potential and corrosion current density extracted from Tafel curves can be used to assess a metal’s corrosion rate. However, such a method is unreliable for Mg alloys since the surface state of Mg alloys changes rapidly and hydrogen evolves quickly when anodic polarization is applied. Studies [26,27] showed that corrosion current density is underestimated when Tafel extrapolation for Mg alloys is performed because of anodic superfluous hydrogen evolution. However, the corrosion current density can be obtained by extrapolating the cathodic region of PDP curves in Figure 4. Compared with AZ91 alloy (1.226 × 10^−5^ A/cm^2^), the corrosion current density (*i*_corr_) of the coating (2.131 × 10^−8^ A/cm^2^) is three orders of magnitude smaller, and the corrosion potential (*E*_corr_) of the coating is 0.16 V nobler, consistent with the OCP measurements. Importantly, the anodic polarization curves for the alloy and the coating exhibit a similar evolution with applying anodic potentials, especially at a small anodic polarization. This similarity originates from the same anodic reaction (i.e., Mg metal is oxidized to Mg^2+^) on the alloy and coated alloy. Thus, it can be deduced that the electrons released by Mg dissolution can be easily captured by the water molecules, or the potentiostat along the passageways of the porous coating under an anodic potential, confirming the high possibility of localized corrosion of the MAO-coated alloy.

**Figure 5:**
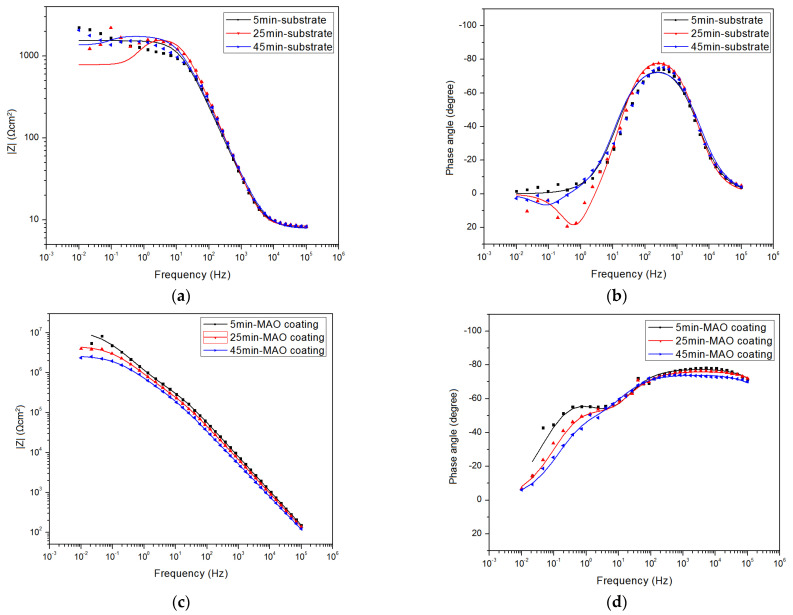
EIS measurements (scattered points) and fitting results (solid lines) of the as-received alloy and the MAO-coated alloy at different immersion times at OCP. (**a**,**c**) Bode plots of impedance vs. frequency and (**b**,**d**) bode plots of phase angle vs. frequency.

Figure 5 shows time-dependent EIS Bode plots for AZ91 alloy and MAO-coated alloy. The scattered points represent raw data, and the solid lines are plotted by fitting data using Zview software (version 3.5). The AZ91 alloy exhibits a different EIS response compared with the coated alloy. Specifically, the EIS response of AZ91 alloy is characterized by a well-defined capacitive loop at high frequency and an inconspicuous inductive loop at low frequency, especially for EIS response after 25 min immersion. The capacitive loop at high frequency represents a single time constant and is generally associated with forming the oxides/hydroxides layer and double electric layer. The apparent inductive loop at low frequency suggests that Mg oxidation occurs due to a rapid Cl^−^ adsorption rate. By comparison, two ill-defined capacitive loops at the high and medium frequencies, and no inductive loop at low frequency, are present in the EIS responses for the MAO-coated alloy. The two capacitive loops represent two time constants, and they are related to the MAO layer and double electric layer. The absence of the inductive loop for the MAO-coated alloy indicates that the adsorption of Cl^−^ is slow due to the thick coating.

The corrosion protection performance can be statistically estimated by low-frequency impedance modulus (|Z|). In Figure 5a,c, the |Z| values decrease with increasing immersion time for both the alloy and the coated alloy. Notably, |Z| values of the alloy are three orders of magnitude smaller than that of the coated alloy, consistent with the PDP results in Figure 4. The biggest |Z| value for AZ91 alloy is only 2.0 × 10^3^ Ω cm^2^, whereas the biggest |Z| for the coating reaches 9.3 × 10^6^ Ω cm^2^ after 5 min immersion. Such a big |Z| of the coating is indicative of excellent corrosion protection for the substrate for 1 h free corrosion.

**Figure 6:**
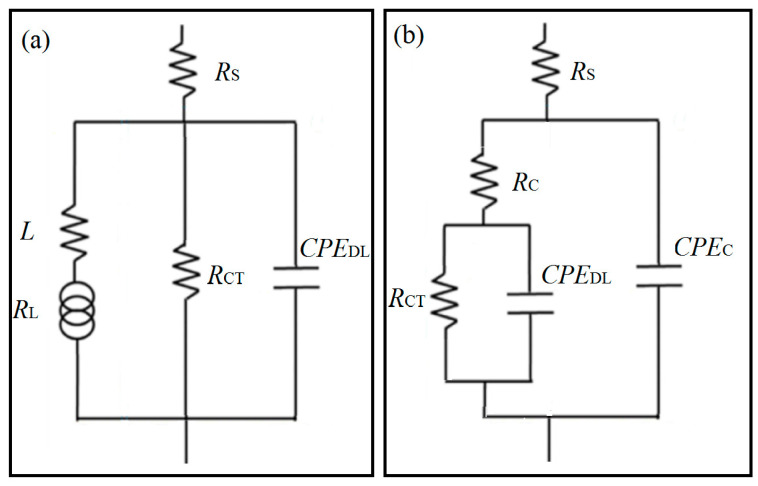
Equivalent electrical circuit proposed to fit the EIS responses of the corrosion systems. (**a**) For alloy and (**b**) for MAO coating.

Figure 6 shows the equivalent electrical circuits used to fit EIS responses. The different profile of EIS responses for the alloy and the coating is the reason for using different equivalent circuits. In the equivalent circuits proposed, constant phase elements (CPE) are used as a substitute for a perfect capacitor since the most negative phase angle in EIS response is not −90°. Besides, *R*_S_ is solution resistance, and *R*_CT_ and *CPE*_DL_ represent charge transfer resistance and double-layer capacitance at the solid/liquid interface, respectively. *R*_L_ and *L* stand for the inductance and resistance associated with inductive loops for AZ91 alloy. *R*_C_ and *CPE*_C_ represent the capacitance and resistance of the MAO-barrier layer. The numerical fitting results of *R*_CT_, *R*_C_, and *R*_L_ are provided in Table 2 (the fitting values of all components are given in Table S1). *R*_ZF_ in Table 2 represents zero-frequency resistance (*f* → 0). It is used to estimate the corrosion rate of AZ91 alloy due to the presence of inductive behavior. The zero-frequency resistance of AZ91 alloy ($R_{\mathrm{ZF}}^{\mathrm{Sub}}$) and MAO coating ($R_{\mathrm{ZF}}^{\mathrm{MAO}}$) can be estimated:

(1) $$R_{\mathrm{ZF}}^{\mathrm{Sub}}=\frac{1}{\frac{1}{R_{\mathrm{CT}}}+\frac{1}{R_{L}}}$$

(2) $$R_{\mathrm{ZF}}^{\mathrm{MAO}}=R_{C}+R_{\mathrm{CT}}$$

From Table 2, $R_{\mathrm{ZF}}^{\mathrm{MAO}}$ is far bigger than $R_{\mathrm{ZF}}^{\mathrm{Sub}}$, consistent with the results in Figure 4 and Figure 5.

### 3.3. Evolution of Corrosion Morphology and Corrosion Products

**Figure 7:**
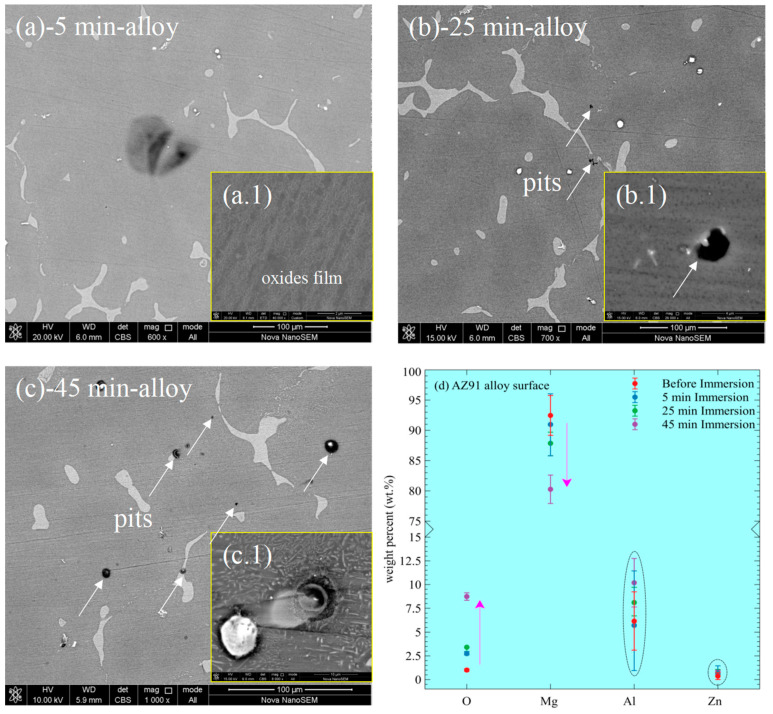
Corrosion morphology of AZ91 alloy and MAO-coated alloy for different immersion times in 3.5% NaCl solution. (**a**–**c**) the alloy surface morphology after 5, 25, and 45 min immersion; (a.1–c.1) the localized corrosion morphology, and (**d**) the weight percent (wt.%) of the elements on the corroded surfaces.

Figure 7 shows the corroded surface of AZ91 alloys before and after MAO treatment at different immersion times in the 3.5% NaCl solution. For AZ91 alloy, there are no evident corrosion features after 5 min immersion in Figure 7a. However, an oxide/hydroxide film forms (Figure 7(a.1)), but the film covers a part of the corroded surface. After 25 min, pits initiate randomly on the corroded surface (Figure 7b). The dissolution of the film and Mg metals is severe on the black corroded sites in Figure 7(b.1). At this moment, corrosion dissolution slightly outweighs the formation of corrosion products (Mg oxides/hydroxides). After 45 min, the number of pits increases with time (Figure 7c), indicating the corrosion aggravates the localized breakdown of the alloy substrate. On some pits, the corrosion products deposit and develop a volcano-like structure in a size of 10–20 μm (Figure 7(c.1)). The elemental compositions (O, Mg, Al, and Zn) of the alloy, before and after corrosion for different immersion times, are compared in Figure 7d. The variation of Al and Zn is small and irrelevant to the occurrence of corrosion. Notably, the alloy exhibits an increasing amount of O and a decreasing amount of Mg with immersion time. The tendency of Mg and O indicates the continuous formation of Mg oxides/hydroxides. However, the amount of O after 25 min approximates that after 5 min, confirming the relatively severe film/alloy dissolution.

**Figure 8:**
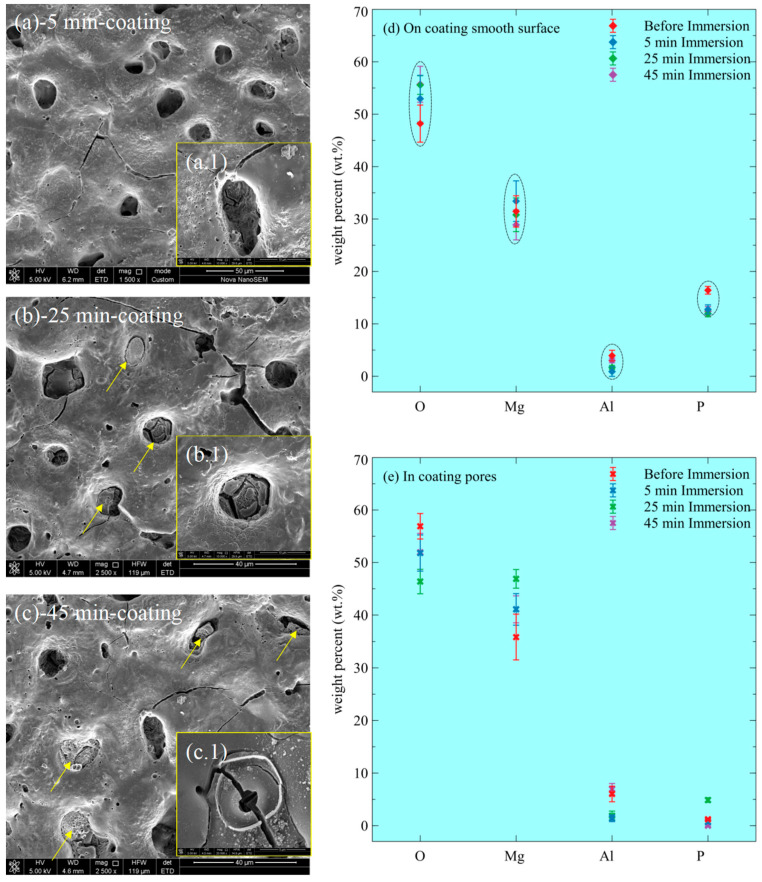
Elemental compositions of corrosion products of AZ91 alloy and MAO-coated alloy at different immersion times in a 3.5% NaCl solution. (**a**–**c**) the coating surface morphology after 5, 25, and 45 min immersion; (a.1–c.1) the localized corrosion morphology; (**d**) the weight percent (wt.%) of the elements on the smooth corroded surface; (**e**) the weight percent (wt.%) of the elements in coating pores.

The corroded surface of the coating after 5 min immersion is characterized by slight chemical damage of oxides/phosphates, resulting in small holes in Figure 8(a.1). After 25 min, some pores are filled with corrosion products (Figure 8b), indicating localized corrosion occurs inside these pores. After 45 min, an increasing amount of corrosion products filled the initial pores in Figure 8c, developing volcano-like structures (Figure 8(c.1)). The coating after immersion shows intact surfaces (the smooth regions), without apparent corrosion morphology, supported by the elemental compositions in Figure 8d that the elements (O, Mg, Al, and P) vary insignificantly. By comparison, the materials inside the pores are under corrosion by the SEM observations and the elemental compositions in Figure 8e. For the corrosion products inside the pores, the amount of Mg increases, indicating the dissolution of the underlying Mg metal and the formation of corrosion products inside the pores. The new-formed corrosion products are mainly Mg oxides/hydroxides because the amount of P is a trifle in Figure 8e.

## 4. Discussion

### 4.1. Corrosion Rate

**Figure 9:**
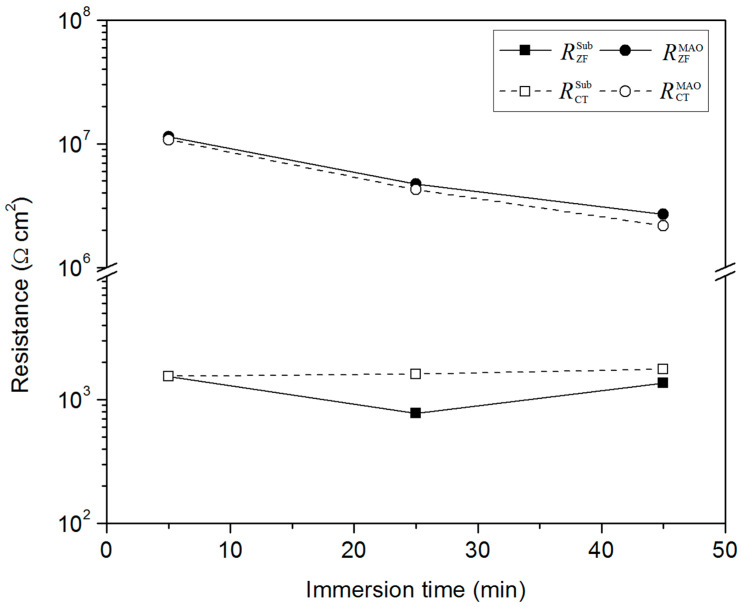
Comparison of *R*_CT_ and *R*_ZF_ of AZ91 alloy and MAO-coated alloy from fitted EIS results at different immersion times in a 3.5% NaCl solution.

The time evolution of the corrosion rate can be evaluated by the resistances (*R*_CT_ and *R*_ZF_) extracted by EIS responses, as shown in Figure 9. The corrosion resistances of MAO-coated alloy far outweigh that of the alloy, consistent with the PDP results in Figure 4. For the alloy in Figure 9, the *R*_CT_ slightly increases with immersion time, indicating a decreasing electron transfer rate from alloy to solution as corrosion proceeds. The thin oxides/hydroxides film (Figure 7(a.1),d), formed continuously with time on the corroded surface, serves as a barrier to slow down the electron transfer rate. However, *R*_ZF_ experiences a decrease from 5 to 25 min, followed by an increase from 25 to 45 min. The smallest *R*_ZF_ at 25 min can be attributed to the rapid dissolution of Mg metal and oxide/hydroxide film (Figure 7b). The dissolution is also responsible for the fluctuation of OCP from 150 to 1800 s (Figure 3). The *R*_ZF_ at 45 min is smaller than that at 5 min, attributing to the presence of inductive behavior (Figure 5c). The inductive behavior is related to Mg metal oxidation at a rapid Cl^−^ adsorption rate, triggering pitting corrosion (Figure 7b). Although the pitting corrosion is more significant after 45 min immersion (Figure 7c) than after 25 min, *R*_ZF_ is bigger due to the thickening corrosion product’s film (Figure 7d) on the corroded surface. Meanwhile, the continuous deposition of corrosion products brings about a relatively stable OCP in Figure 3.

For the coated alloy, it is interesting that the MAO-coated alloy exhibits high resistance in the case that the coating has considerable pores and cracks. Firstly, the 25–30 μm thick phosphate/oxide ceramic composites stifle uniform chemical dissolution. Secondly, at the early-stage immersion, although corrosion of the underlying Mg alloy proceeds through the through-pores, the electron transfer rate from alloy to solution is limited spatially due to the thick coating, and thus, the charge transfer resistance is very big. Moreover, the through-pores are isolated before coating ruptures in a large area at the early-stage immersion. From Figure 9, *R*_CT_ and *R*_ZF_ decrease with immersion time, indicating an increased corrosion rate. The isolated through-pores offer passageways for corrosive media penetrating the underlying alloy. As a result, corrosion occurs inside the pores to deteriorate the protection of the coating (Figure 8(b.1,c.1)). Accordingly, the localized corrosion of the underlying alloy accelerates with time, but the overall corrosion rate of the coating is relatively small, and the coating is still intact from the surface appearance (the smooth regions in Figure 8).

### 4.2. Corrosion Behavior

#### 4.2.1. Mg-Based Alloys

Mg and its alloy are very active and ready to dissolve when exposed to corrosive media, leading to the local formation of Mg oxides/hydroxides film on the corroded surface accompanied by hydrogen evolution. The anodic and cathodic reactions of Mg and its alloys are as follows:

(3) $$\mathrm{Anodic}\;\mathrm{reaction}:\;\mathrm{Mg}↔{\mathrm{Mg}}^{2+}+2e^{-}$$

(4) $$\mathrm{Cathodic}\;\mathrm{reaction}:\;2H_{2}O+2e^{-}↔2{\mathrm{OH}}^{-}+H_{2}$$

(5) $$\mathrm{Overall}\;\mathrm{reaction}:\;\mathrm{Mg}+{2H}_{2}O↔\mathrm{Mg}{\left(\mathrm{OH}\right)}_{2}+H_{2}$$

The corrosion products of Mg(OH)_2_ are always formed at a relatively high OH^−^ in the solution. The relationship between [Mg^2+^] and pH to assess the thermodynamic stability of Mg(OH)_2_ is as follows:

(6) $$\log[{\mathrm{Mg}}^{2+}]=17.26-2\mathrm{pH}$$

The [Mg^2+^] and pH in the bulk solution after corrosion for different times in the 3.5% NaCl solution are given in Table 3, based on the ICP-OES measurements.

According to equation six, the formation of an Mg(OH)_2_ film is impossible at the macroscopic level using the data of [Mg^2+^], and pH in Table 3. This is the essential reason for the large area of the exposed alloy without covering by oxides/hydroxides after immersion in Figure 7. On the other hand, the dissolution of the metal and oxides/hydroxides prevail due to the presence of the Cl^−^ ions by reaction (7):

(7) $$\mathrm{Mg}{\left(\mathrm{OH}\right)}_{2}(s)+{2\mathrm{Cl}}^{-}(\mathrm{aq})↔{\mathrm{MgCl}}_{2}(\mathrm{aq})+2{\mathrm{OH}}^{-}(\mathrm{aq})$$

The Cl^−^ ions also contribute to the pitting corrosion of the alloy in Figure 7b,c after 25 and 45 min immersion.

**Figure 10:**
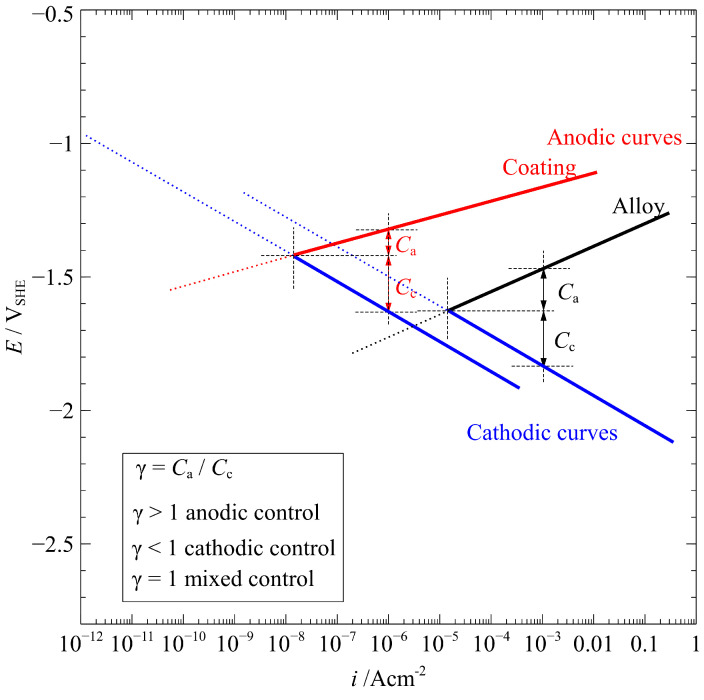
An ideal schematic mechanism of anodic and cathodic curves for AZ91 alloy and MAO-coated alloy after 1 h immersion in a 3.5% NaCl.

Theoretically, metal corrosion can be classified as being under anodic, cathodic, or mixed control. If the metal is under cathodic control, small changes in current density result in larger changes in cathodic potential than in anodic potential [6]. In order to evaluate the corrosion-control reaction, an ideal schematic mechanism of anodic and cathodic curves for the alloy after 1 h corrosion is shown in Figure 10. At the macroscopic level, it is revealed that corrosion of the AZ91 alloy is under cathodic control (γ = *C*_a_/*C*_c_ < 1) for the 1 h immersion corrosion in a 3.5 % NaCl solution. However, at the microscopic level, pit corrosion initiates due to the attack of Cl^−^ on the bared Mg metal. Subsequently, oxides/hydroxides form on the pits, developing volcanic-like structures. The deposition of oxides/hydroxides indicates the locally high [Mg^2+^] and pH on the corroded area. In this case, the Mg oxidization reaction controls the electrochemical corrosion, suggesting an anodic control. Moreover, the volcanic-like structures slow down the propagation rate of pitting corrosion, possibly because the chemical reaction (7) proceeds from products to reactants at a very high pH.

Overall, the corrosion of the AZ91 alloy in a 3.5% NaCl solution is generally under cathodic control since the Mg oxidation reaction proceeds freely without obstruction from oxides/hydroxides film, leading to quasi-uniform corrosion. Although pitting corrosion initiates and propagates with time, the local formation of corrosion products makes the pitting corrosion seem insignificant to the overall damage.

#### 4.2.2. MAO-Coated Alloy

In the presence of Cl^−^, the coated alloy is initially subjected to a chemical attack (Figure 8a). For 5 min immersion, OCP that experiences an instant decrease and subsequent rebound (Figure 3) is indicative of the chemical dissolution of the coating materials. However, Mg^2+^ in the bulk solution after 1 h immersion cannot be detected by ICP–OES measurements in this study and the pH experiences no changes with immersion time, meaning that the chemical decomposition is inappreciable. The MAO-coated alloy shares the same electrochemical reactions (3–5) with respect to AZ91 alloy, so the evolution and profile of OCP and PDP are similar except for the values. The thermodynamic similarity decides the same cathodic control (γ =*C*_a_/*C*_c_ < 1 in Figure 10) for the coating in the early-stage immersion corrosion.

The through-pores-induced localized corrosion makes a decisive contribution to the corrosion current density for the coating in Figure 4. Before coating ruptures in a large area, these isolated pores can be treated as the existing pits, where Cl^−^ ions and water molecules can infiltrate easily. As a result, decompositions of the oxides/phosphates in the pores bring about the direct contact of the underlying alloy with the aggressive solution and the subsequent pitting corrosion initiation. Thus, electrochemical reactions occur, forming high [Mg^2+^] and pH in the micro sized pores. Then, the formation of Mg oxides/hydroxides is ready, theoretically slowing down the further electrochemical corrosion of the underlying alloy. However, the corrosion resistance (Figure 9) of the coating decreases with time because the newly formed oxides/hydroxides (corrosion products) inside the pores are not compact (as shown by the large cracks in Figure 8(b.1,c.1)). Additionally, in the deep pores (deeper than the pits for the uncoated alloy), the local concentration of Cl^−^ ions may be much bigger than in the bulk solution, leading to the rapidly-forward chemical reaction (7). Thus, more and more pits initiate under the isolated through-pores. With time, pits inside these isolated pores propagate without resistance from the corrosion products, leading to the amalgamation of isolated pores and finally the large-area breakdown of the coating, as we have observed in our previous study [8].

Overall, the corrosion of the coated alloy in a 3.5% NaCl solution for 1 h is also under cathodic control. Electrochemical corrosion of the underlying alloy proceeds continuously, contributing to the increased corrosion rate with time.

## 5. Conclusions

The AZ91 Mg alloy was treated by micro-arc oxidation in the phosphate-based electrolyte, and the early-stage electrochemical corrosion behavior of the alloy before and after treatment was investigated. The coating has a typical MAO structure characterized by cracks and various pores that are vulnerable sites to trigger corrosion of the underlying alloy. The profiles of OCP and PDP curves of the alloy and coating are similar, but the EIS responses are different. The corrosion resistances extracted from EIS responses for the MAO-coated alloy are three orders of magnitude bigger than that for the uncoated alloy due to the cathodic coating, consistent with the corrosion current density by PDP. With immersion, the corrosion rate increases for the coated alloy. An increasing number of pits initiates on the alloy with time, but they are subsequently covered by corrosion products, which hinders their propagation. The isolated through-pores are like the existing pits on the coating. The incompact corrosion products inside the through-pores are easily damaged, causing more pit initiation and propagation. Accordingly, the electrochemical corrosion of the underlying alloy via the through-pores brings about increasing corrosion rates with time. Overall, electrochemical corrosion for both the alloy and the coated alloy during 1 h immersion in a 3.5% NaCl solution is under cathodic control, leading to quasi-uniform corrosion for the uncoated alloy, and localized corrosion for the coating.

## Figures and Tables

**Table 1:** Chemical compositions (wt.%) of AZ91 Mg alloy.

Al	Zn	Mn	Si	Cu	Ni	Fe	Mg
8.5–9.3	0.4–1.0	0.15	0.1	0.03	0.002	0.005	Bal.

**Table 2:** The fitting results of resistances in the EEC for AZ91 alloys before and after MAO treatment after different immersion times.

t/min	*R*_CT_ (Ω cm^2^)		*R*_L_ (Ω cm^2^)	*R*_C_ (Ω cm^2^)	*R*_ZF_ (Ω cm^2^)	
	Alloy	Coating	Alloy	Coating	Alloy	Coating
5	1650 (10.6)	1.100 × 10^7^ (27.9)	2.706 × 10^5^ (33.9)	9.500 × 10^5^ (39.1)	1640 (13.6)	1.195 × 10^7^ (49.3)
25	1710 (14.1)	1.193 × 10^6^ (38.9)	1501 (12.6)	6.280 × 10^5^ (43.8)	799.3 (6.62)	4.821 × 10^6^ (28.6)
45	1830 (22.3)	2.174 × 10^6^ (21.4)	6542 (36.2)	5.820 × 10^5^ (42.4)	1430 (27.4)	2.756 × 10^6^ (39.2)

The values in parentheses represent the standard deviations from the three measurements.

**Table 3:** [Mg^2+^] and pH in the bulk solution for AZ91 alloy after immersion at different times.

**t/min**	**5**	**15**	**25**	**35**	**45**	**60**
[Mg^2+^]	6.502 × 10^−6^	6.8313 × 10^−6^	7.2428 × 10^−6^	7.8601 × 10^−6^	8.0247 × 10^−6^	8.7243 × 10^−6^
pH	7.04	7.11	7.12	7.24	7.38	7.42

## Data Availability

The data presented in this study are available on request from the corresponding author.

## Supplementary Materials

The following supporting information can be downloaded at: https://www.mdpi.com/article/10.3390/ma15227909/s1, Table S1: AZ91 alloy before and after MAO treatment: fitting results from EIS measurements in 3.5% NaCl solution.
