# Supplementary material for: A Study of Early-Stage Corrosion Behavior of AZ91 Alloy and MAO-Coated Alloy in 3.5% NaCl Solutions
Source: Materials (Basel). 2022 Nov 9;15(22):7909. doi: 10.3390/ma15227909 (PMC9699487; doi:10.3390/ma15227909)
Supplement: Supplementary file 1 [file materials-15-07909-s001.zip › materials-1965719-supplementary.pdf]

Supplementary Material

Table S1. AZ91 alloy before and after MAO treatment: fitting results from EIS measurements in 3.5% NaCl solution.

	**Time (min)**	**R_S (Ωcm^2)**	**R_C (Ωcm^2)**	**R_{CT} (Ωcm^2)**	**CPE_{DL} × 10^{-5} ($\Omega^{-1}cm^{-2}s^n$)**	**n_{DL}**	**CPE_C × 10^{-5} ($\Omega^{-1}cm^{-2}s^n$)**	**n_{DL}**	**R_L (Ωcm^2)**	**L (Hcm^{-2})**	**R_{ZF} (Ωcm^2)**	**χ^2 (Chi-Squared)**
AZ91 alloy	5	22.57 (2.304)	/	1650 (4.767)	2.885 (9.056)	0.930 (4.76)	/	/	2.706×10^5 (11.8)	7.177×10^4 (8.294)	1640	1.004×10^{-1}
	25	23.87 (0.528)	/	1710 (1.757)	3.131 (4.720)	0.885 (1.074)	/	/	1.501×10^3 (15.67)	1.599×10^5 (14.39)	799.3	5.429×10^{-3}
	45	22.20 (0.981)	/	1830 (2.832)	5.163 (8.024)	0.797 (3.331)	/	/	9.326×10^3 (14.21)	3.120×10^4 (11.18)	1430	4.931×10^{-2}
MAO-coated alloy	5	26.33 (1.761)	9.500×10^5 (11.747)	1.1×10^7 (1.704)	0.135 (3.204)	0.599 (1.155)	0.521 (4.034)	1	/	/	1.195×10^7	6.668×10^{-3}
	25	24.26 (2.461)	6.280×10^5 (9.249)	1.193×10^6 (3.118)	0.237 (3.651)	0.649 (1.405)	0.646 (3.183)	1	/	/	4.821×10^6	3.224×10^{-3}
	45	19.32 (1.212)	5.820×10^5 (5.019)	2.174×10^6 (2.935)	0.201 (6.762)	0.661 (2.934)	0.601 (3.941)	1	/	/	2.756×10^6	7.273×10^{-4}

The values in parentheses represent the fitting error (%) using Zview software.
